# Supplementary material for: Haplotype Structures and Protein Levels of TGFB1 in HPV Infection and Cervical Lesion: A Case-Control Study
Source: Cells. 2022 Dec 25;12(1):84. doi: 10.3390/cells12010084 (PMC9818366; doi:10.3390/cells12010084)
Supplement: Supplementary file 1 [file cells-12-00084-s001.zip › cells-2087347-supplementary.pdf]

Supplementary Materials

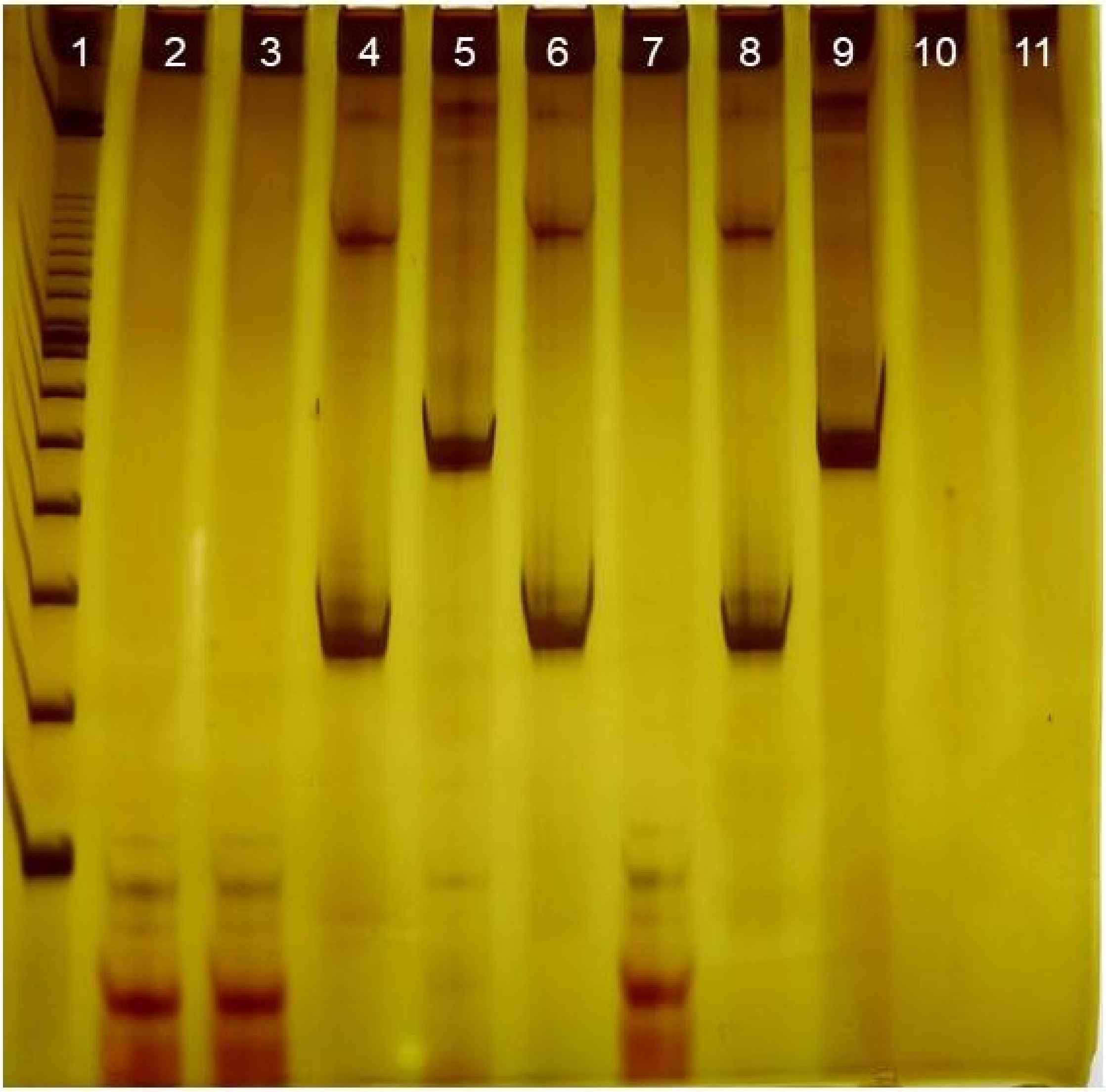

Figure S1: Electrophoresis gel image for HPV detection by PCR. The concomitant amplification of the human B-globin gene fragment (268 bp) and the HPV DNA fragment (~450 bp) for the same sample of DNA extracted from cervical cells confers the status of HPV-infected on the sample donor. Amplification only of the B-globin fragment and not of the HPV confers the HPV-uninfected status. Columns: 1 – marker ladder (100 bp); 2 and 3 – negative control for HPV amplification; 4 and 5 – positive control for HPV detection; 6 and 7 – HPV-uninfected patient; 8 and 9 – HPV-infected patient; 10 and 11 – negative control for B-globin amplification.

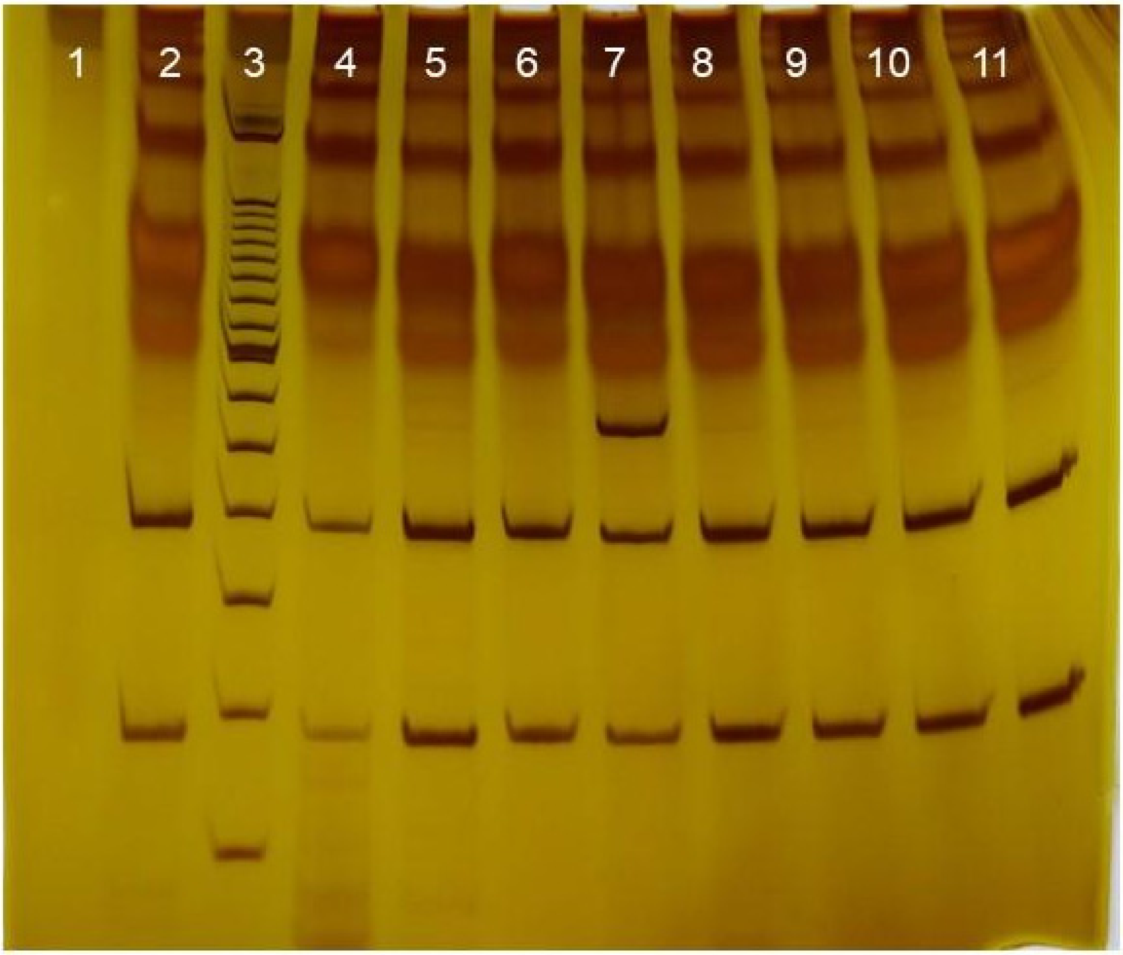

Figure S2: Electrophoresis gel image for *TGFB1* rs1800468 (c.–1638G>A) genotyping. PCR products (597 bp) digested by restriction enzyme *HpyCH4IV*. Columns: 1 – not loaded; 2, 4, 5, 6, 8, 9, 10 and 11 – GG genotype, showing fragments of 402 bp and 195 bp; 7 – GA genotype, showing fragments of 597 bp, 402 bp and 195 bp; 3 - marker ladder (100 bp).

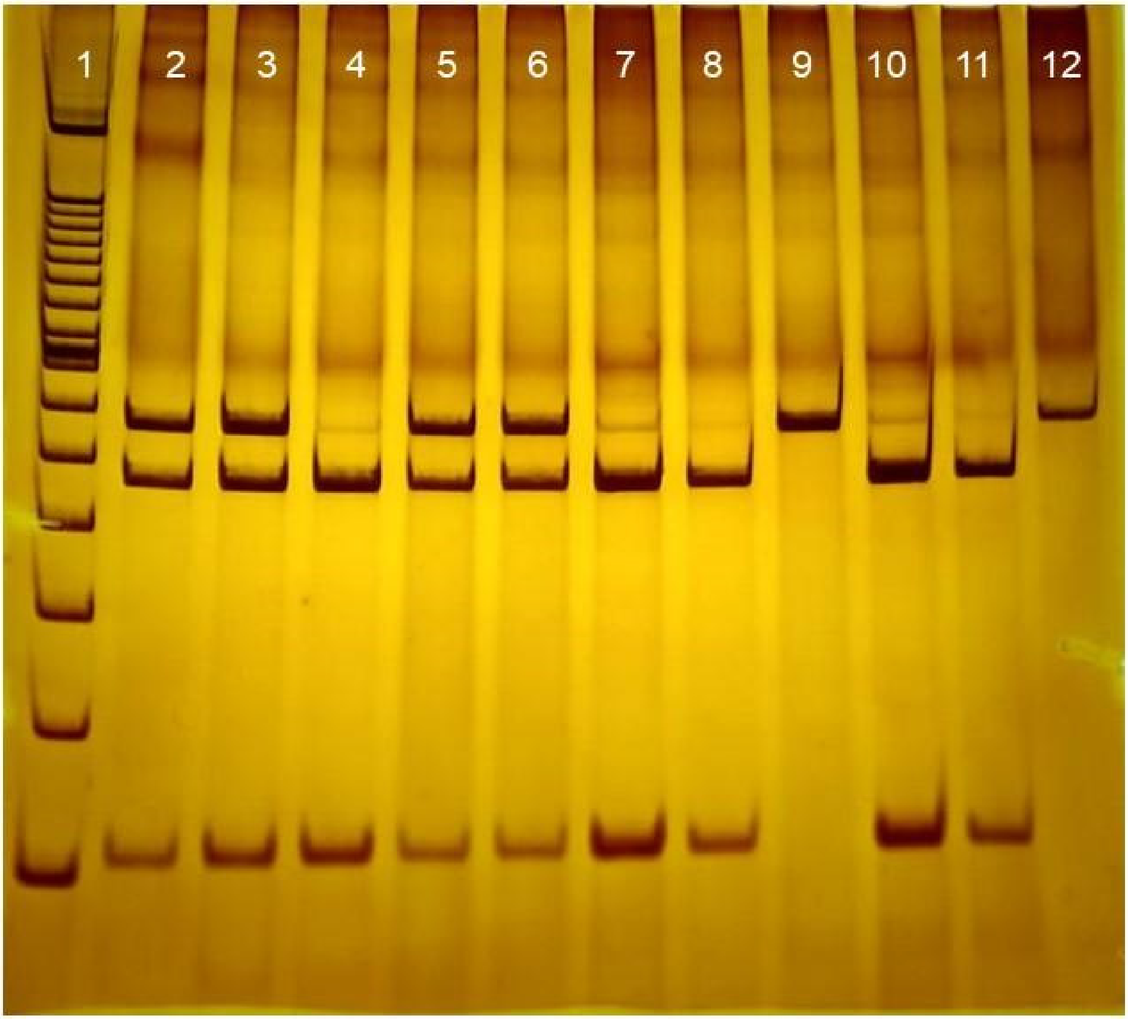

Figure S3: Electrophoresis gel image for *TGFB1* rs1800469 (c.–1347C>T) genotyping. PCR products (597 bp) digested by restriction enzyme *Bsu36I*. Columns: 1 – marker ladder (100 bp); 2, 3, 5 and 6 – CT genotype, showing fragments of 597 bp, 488 bp and 109 bp; 4, 7, 8, 9 and 10 – CC genotype, showing fragments of 488 bp and 109 bp; 9 and 12 – TT genotype, showing fragments of 597 bp.

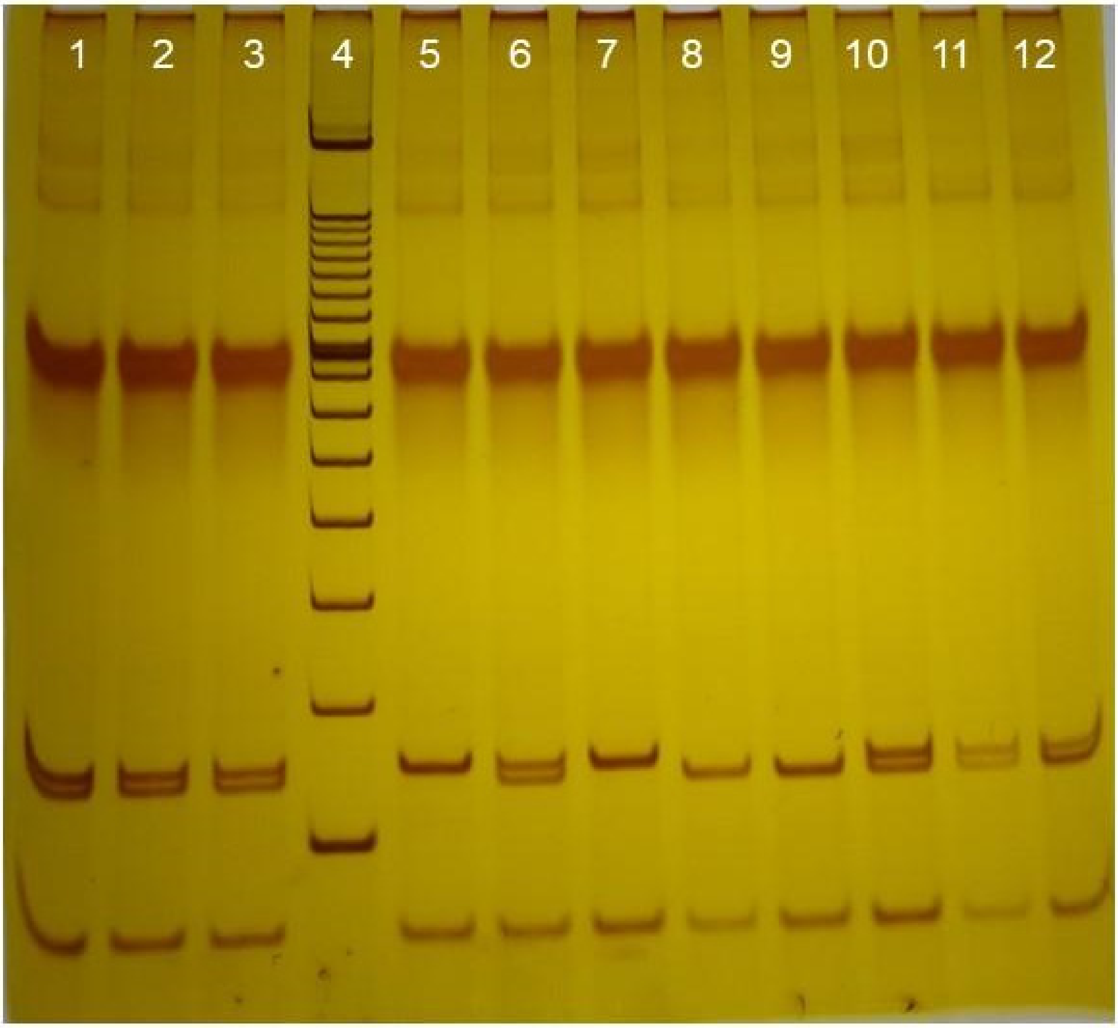

Figure S4: Electrophoresis gel image for *TGFB1* rs1800470 (c.29C>T) genotyping. PCR products (294 bp) digested by restriction enzyme *MspA1I*. Columns: 1, 2, 3, 6, 10, 11 and 12 – CT genotype, showing fragments of 161 bp, 149 bp and 67 bp; 5 and 7 – TT genotype, showing fragments of 161 bp and 67 bp; 8 and 9 – CC genotype, showing fragments of 149 bp and 67 bp; 4 – marker ladder (100 bp). Note: fragments smaller than 67 bp came off the bottom edge of the gel during electrophoresis.

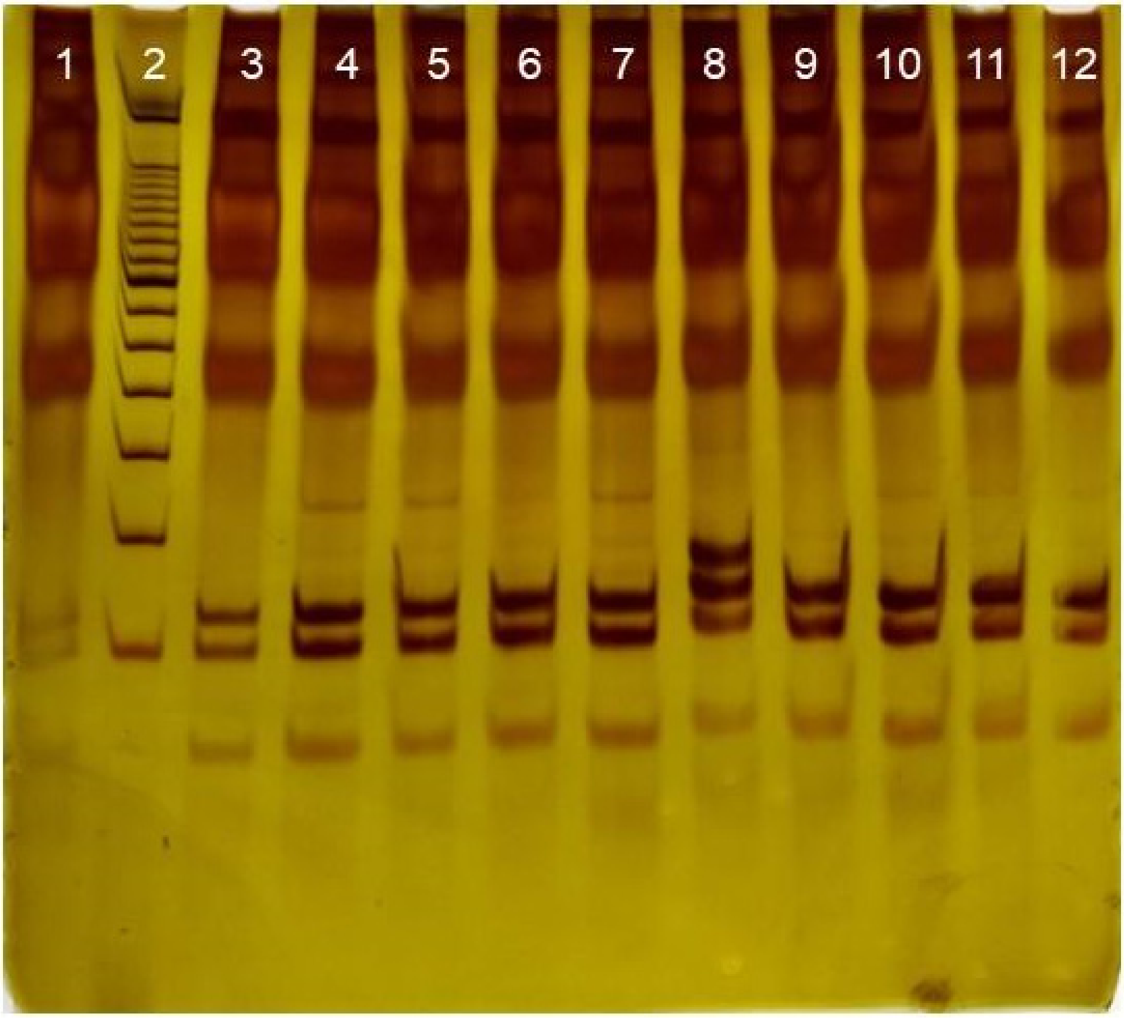

Figure S5: Electrophoresis gel image for *TGFB1* rs1800471 (c.74G>C) genotyping. PCR products (294 bp) digested by restriction enzyme *BglI*. Columns: 1, 3, 4, 5, 6, 7, 9, 10, 11 and 12 – GG genotype, showing fragments of 131 bp, 103 bp and 60 bp; 8 – GC genotype, showing fragments of 163 bp, 131 bp, 103 bp and 60; 2 – marker ladder (100 bp).
